# Long-term exposure to 835 MHz RF-EMF induces hyperactivity, autophagy and demyelination in the cortical neurons of mice

**DOI:** 10.1038/srep41129

**Published:** 2017-01-20

**Authors:** Ju Hwan Kim, Da-Hyeon Yu, Yang Hoon Huh, Eun Ho Lee, Hyung-Gun Kim, Hak Rim Kim

**Affiliations:** 1 Department of Pharmacology, College of Medicine, Dankook University, Cheonan-si, Chungnam, Republic of Korea; 2 Center for Electron Microscopy Research, Korea Basic Science Institute, Ochang, Chung-Buk, Republic of Korea

## Abstract

Radiofrequency electromagnetic field (RF-EMF) is used globally in conjunction with mobile communications. There are public concerns of the perceived deleterious biological consequences of RF-EMF exposure. This study assessed neuronal effects of RF-EMF on the cerebral cortex of the mouse brain as a proxy for cranial exposure during mobile phone use. C57BL/6 mice were exposed to 835 MHz RF-EMF at a specific absorption rate (SAR) of 4.0 W/kg for 5 hours/day during 12 weeks. The aim was to examine activation of autophagy pathway in the cerebral cortex, a brain region that is located relatively externally. Induction of autophagy genes and production of proteins including LC3B-II and Beclin1 were increased and accumulation of autolysosome was observed in neuronal cell bodies. However, proapoptotic factor Bax was down-regulted in the cerebral cortex. Importantly, we found that RF-EMF exposure led to myelin sheath damage and mice displayed hyperactivity-like behaviour. The data suggest that autophagy may act as a protective pathway for the neuronal cell bodies in the cerebral cortex during radiofrequency exposure. The observations that neuronal cell bodies remained structurally stable but demyelination was induced in cortical neurons following prolonged RF-EMF suggests a potential cause of neurological or neurobehavioural disorders.

Wireless mobile phone communication is globally ubiquitous and popular. There have long been concerns regarding possible adverse biologically-related health effects of exposure to radiofrequency electromagnetic field (RF-EMF). The central nervous system (CNS) is a main concern with regards to the effects of RF-EMF, since mobile phone use involves close exposure or immediate contact with the head^1^. The biological effects of RF-EMF exposure on human health remain unclear because of conflicting findings of various studies^2^,^3^.

A number of studies have reported that RF-EMF exposure of animal models increases blood-brain barrier permeability, impairs intracellular calcium homeostasis, alters neurotransmitters, and increases neuronal loss and damage in brain tissue^4^,^5^,^6^,^7^,^8^. Epidemiologic studies have linked RF-EMF exposure from mobile phones with neurological and cognitive dysfunctions^9^,^10^,^11^.

Cellular effects of RF-EMF exposure reportedly involve the apoptotic pathway, extracellular signal pathway, DNA damage response, cell proliferation, and cell cycle^3^,^12^,^13^,^14^,^15^. The effect of EMF exposure on autophagy in mammalian cells has been documented^16^,^17^. Autophagy is catabolic cellular degradation process responsible for degrading damaged organelles or unusual protein aggregates, which is activated in the presence of a variety of stimuli^18^. Suppression of autophagy may have a role in progression of cancers, neurodegenerative diseases, and infections, and is a common feature of aging^19^,^20^. Therefore, autophagy plays an important role in maintaining cellular homeostasis and further functions protecting cells from various stressors^21^.

The cerebral cortex is a thin layer of neural tissue^22^ that surrounds brain tissues such as hippocampus, striatum, basal ganglia, and thalamus. In addition, the mouse cortex has a smooth surface, while that of humans is folder rather like a walnut^23^. It is a highly-developed region of the human brain that processes most of the actual information, including sensory functions, such as hearing, touch, vision, smell, and movement, as well as cognitive functions, such as thought, perception, memory-related problem solving, and understanding language^24^,^25^. Abnormalities of the human cerebral cortical region could be associated with various neurodegenerative diseases including Alzheimer’s disease, Lafora disease, and various cognitive disorders^26^,^27^,^28^. RF-EMF exposure of the human cerebral cortex reportedly causes physiological alterations in blood flow and increases glucose metabolism^29^,^30^. Exposure of cultured neurons to RF-EMF results in neurotoxicity, with oxidative damage caused to mitochondrial DNA^31^. Thus, RF-EMF exposure could induce various neurological changes. Information of phenotypes or symptoms following EMF exposure is still lacking even though some of studies have been reported with respect to electromagnetic hypersensitivity following EMF exposure^32^,^33^.

The present study hypothesized that the cerebral cortex of brain can be appreciably affected by RF-EMF exposure. The focus was cerebral cortical neurons, which are involved in the autophagy intracellular pathway that could function in adaptation to continuous RF-EMF stress or for neuronal protection by generating autophagosome in their neuronal cell bodies. Previously, we showed that RF-EMF exposure induces autophagy in specific interior regions of mice brain^16^. In this study, we have focused on the cerebral cortex of mice. Although the whole body of mice were exposed by RF-EMF system but the cerebral cortex of mice brain is more directly exposed than the interior regions of mice brain because the horn antenna was located top of the exposure cage.

## Results

### Long-term exposure to 835 MHz RF-EMF induces hyperactivity

**Figure 1:**
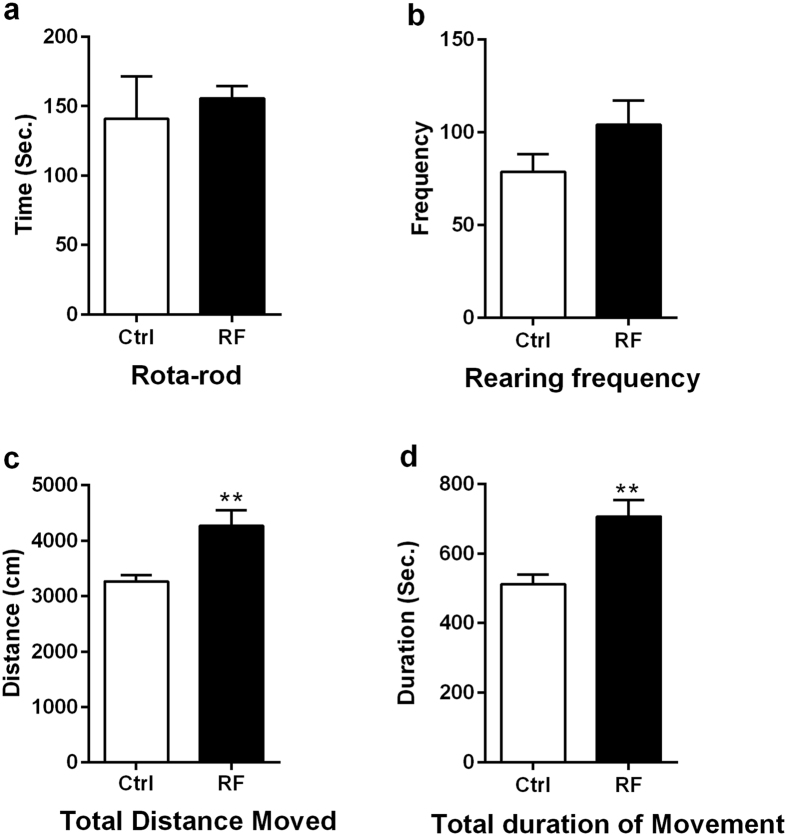
Behavioural tests of RF-EMF exposed mice. Basic motor activity (rota-rod, **a**) and general locomotor activity (rearing frequency, total distance moved, and total duration movement) in the open field (**b**–**d**) were measured after RF-EMF exposure. Each bar illuminates the mean ± SEM of value of 6 mice. *P < 0.05.

The rota rod test was done to determine the impact of chronic RF-EMF exposure on behavioural changes. This test is widely used to evaluate motor dysfunctions, especially coordination and balance. There was no significant difference between the control and RM-EMF groups (Fig. 1a). The latency to fall for control group was 141.0 ± 30.55 and the RF-EMF group 155.7 ± 8.78. Mice were also evaluated using an open field test. This test is usually used to evaluate fear in response to novelty and locomotory motivation^34^. Rearing frequency, total distance moved, and total duration movement were monitored for 30 minutes, with data presented as cumulative total values of each parameter. The total moving distance was significantly increased in the RF-EMF exposed group (4274 ± 280.8) compared to the control group (3265 ± 116.8) (Fig. 1c). Total duration movement in the RF-EMF exposed group was significantly increased compared with the control group (Fig. 1d). However, there is no significant difference in rearing frequencies between the groups (Fig. 1b). Overall, the mice exposed to RF-EMF were hyperactive.

### Autophagy-related genes are significantly up-regulated in the cerebral cortex following RF-EMF exposure

**Figure 2:**
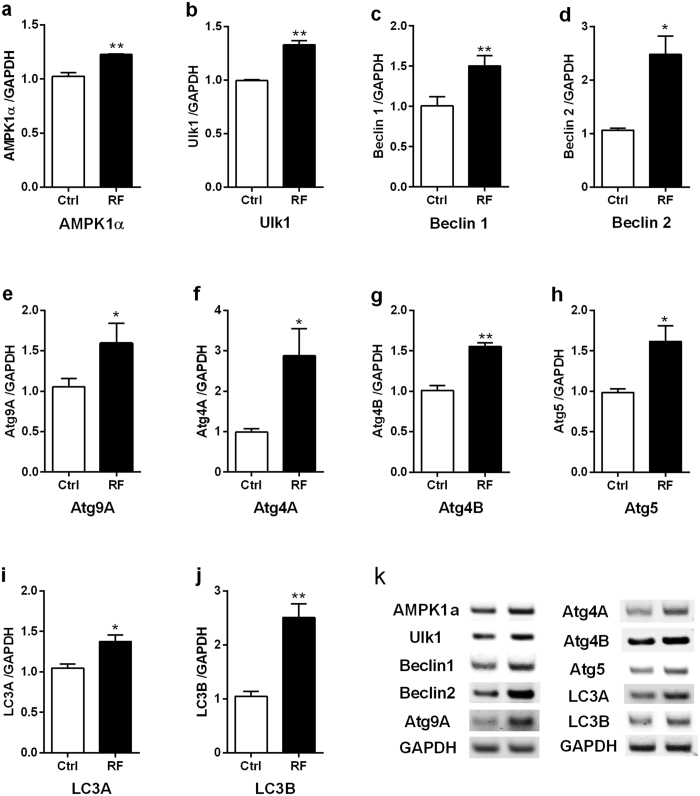
Expressional levels of autophagy related genes in the cerebral cortex of mice following the chronic RF-EMF exposure. The cerebral cortical RNA extracted sham-exposed and RF-exposed mice were analysed for the expression level of autophagy genes by quantitative real-time PCR. (**a**–**j**) Quantification of AMPK1α, Ulk1, Atg4/B, Beclin1/2, Atg5, Atg9A, and LC3A/B mRNA transcripts by qRT-PCR. (**k**) Agarose gel electrophoresis showing differential expression of autophagy genes by sqRT-PCR. The expression values of the cerebral cortex of RF-exposed mice were normalized to those of the sham-exposed mice. The relative mRNA levels of each gene were calculated by normalizing to expression of GAPDH by the 2^−ΔΔCt^ method (n = 6). Each bar represents the mean ± SEM of three independent experiments. Statistical significance was evaluated using a *t*-test: *P < 0.05, **P < 0.01.

To examine if autophagy can be induced in the cerebral cortex of mice following RF-EMF exposure, specific autophagy-related genes were selected and analysed using quantitative real-time PCR and semi-quantitative reverse-transcriptase PCR during the sham or RF-EMF exposure. Autophagy-related genes (AMPK1α, Ulk1, Beclin1/2, Atg9A, Atg4A/B, Atg5, and LC3A/B), which play a key role in formation of autophagy structure in response to various cellular stressors^35^, displayed significantly augmented expression in qPCR (in response to chronic RF-EMF exposure (Fig. 2). Expression of Beclin 1, Atg9A, Atg4B, Atg5, and LC3A mRNA were increased about 1.5 fold (Fig. 2c,e,g,h and i, and Table 1) and the level of Beclin 2, Atg4A, and LC3B transcripts were elevated by 2–2.5 fold (Fig. 2d,f and j, and Table 1) compared to sham-exposed control mice. Semi-quantitative analysis confirmed the qRT-PCR results (Fig. 2k).

### LC3B-II and Beclin1 expression are significantly increased in the cerebral cortex following RF-EMF exposure

**Figure 3:**
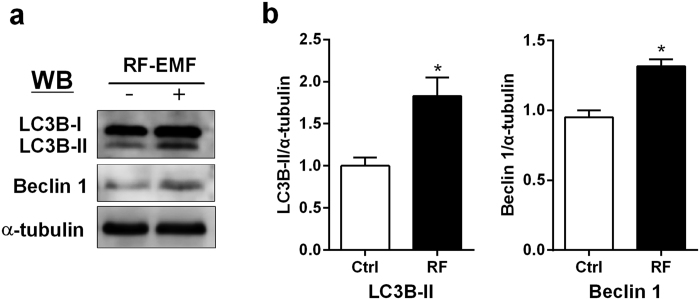
Protein expression of LC3B-II and Beclin1 in the cerebral cortex of mice after chronic RF-EMF exposure. (**a**) Total protein extracted from cerebral cortex of mice was subjected to 10–15% SDS–PAGE and immunoblotted with antibody against LC3B-II and Beclin1. α-tubulin was used as the internal loading control. (**b**) The intensity of bands of western blot was quantified by densitometry. The protein level was normalized relative to α-tubulin. Each bar shows mean of three independent experiments with SEM. Statistical significance was evaluated using two tailed *t*-test: *P < 0.05.

To validate the result of gene profiles from RT-PCR, the expression levels of autophagy proteins were determined using immunoblotting with anti-LC3B-II antibody and anti-Beclin1 antibody in the cerebral cortical lysates from brains following the 12-week sham or RF exposure. The anti-LC3B antibody (Cell Signaling Technology, USA) can detect both 16 kDa LC3B-I and 14 kDa LC3B-II protein. Additionally, to detect LC3B-II protein, high concentration of total protein lysates prepared from cerebral cortex was analysed by Western blot. The immunoblot analysis indicated that the protein levels of both LC3B-II and Beclin1 significantly increased in the cerebral cortex of mice after RF-EMF exposure (Fig. 3). The increased expression of autophagy-related proteins in parallel with the augmented level of LC3B and Beclin1 gene in the cerebral cortex indicating that autophagy was regulated in the level of gene expression and protein expression in the cerebral cortex during the chronic RF-EMF exposure.

### Apoptotic factors Bcl2 and Bax down-regulated in the RF-EMF exposed cerebral cortex

**Figure 4:**
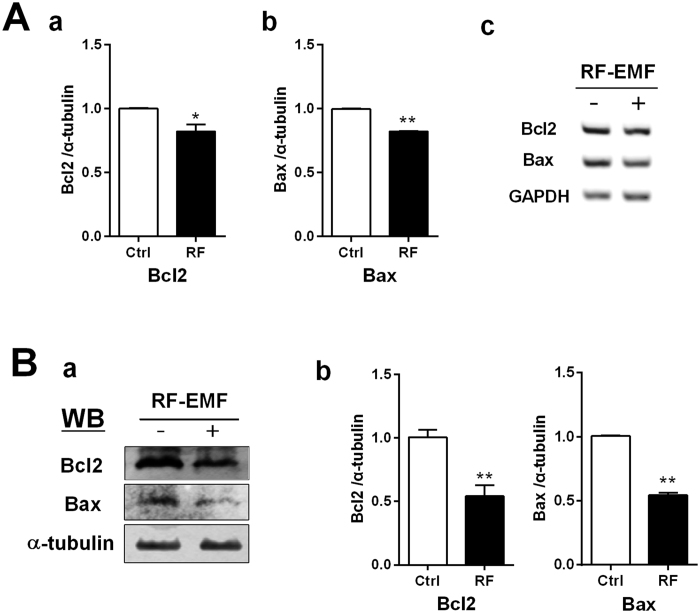
Expression of apoptosis-related genes in the cerebral cortex of mice following chronic RF-EMF exposure. (**A**) The cerebral cortical RNA extracted sham-exposed and RF-exposed mice were analysed for the expression level of autophagy genes by quantitative real-time PCR. (a,b) Quantification of Bcl2 and Bax mRNA transcripts by qRT-PCR. (c) 1.5% Agarose gel electrophoresis showing differential expression of Bcl2 and Bax by sqRT-PCR. The expression values of the cerebral cortex of RF-exposed mice were normalized to those of the sham-exposed mice. The relative mRNA levels of each gene were calculated by normalizing to expression of GAPDH by the 2^−ΔΔCt^ method (n = 6). (**B**) (a) Total protein was subjected to 15% SDS–PAGE and immunoblotted with antibodies against Bcl2 and Bax. α-tubulin was used as loading control. (b) The intensity of bands of western blot was quantified by densitometry. The protein level was normalized relative to α-tubulin. Each bar represents the mean ± SEM of three independent experiments. Statistical significance was evaluated using a *t*-test: *P < 0.05, **P < 0.01.

To determine whether apoptosis was activated or not during the activation of autophagy, the level of the apoptosis component genes Bcl2 (anti-apoptotic) and Bax (pro-apoptotic) genes in mice cerebral cortex were analysed by RT-PCR. qPCR revealed slightly decreased activity of Bcl2 and Bax (Fig. 4A) and immunoblot also showed down-regulation of Bcl2 and Bax protein level (Fig. 4B).

### Accumulation of autophagy in the cerebral cortical neuron after RF-EMF exposure

**Figure 5:**
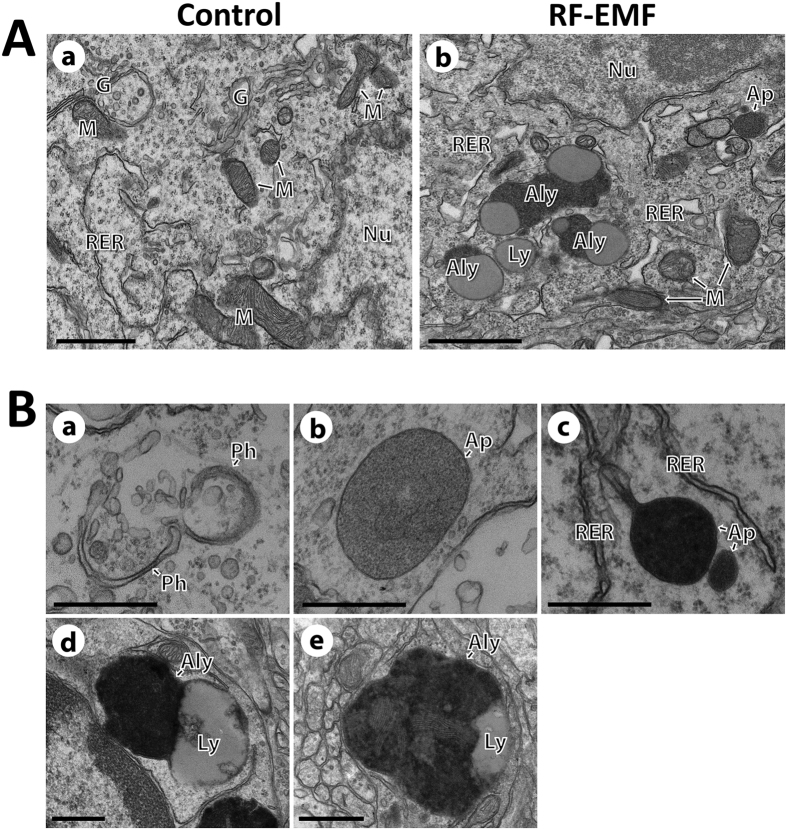
Representative TEM images showing the autophagy in the neuronal cell body of the cerebral cortex following chronic RF-EMF exposure. (**A**) Ultrastructural comparison of autophagy between control and RF-exposed mice. Representative TEM micrographs were acquired from sham control (a) and RF-exposed mice for 12 weeks (b), respectively. In RF-exposed mice, numerous autophagosome (Ap) and autolysosome (Aly) were observed in difference with its sham control. (**B**) A series of autophagic flux process in cerebral cortical neurons after RF-EMF exposure for 12 weeks. (a) Phagophore containing fragments of cytoplasmic organelles; (b) Early autophagosome; (c) Late autophagosome; (d) Early Autolysosome; and (e) Late autolysosome. Abbreviations are: Ap, autophagosome; Aly, autolysosome; G, Golgi apparatus; M, mitochondria; N, nucleus; Ph, phagophore; RER, rough endoplasmic reticulum. Size bars: 1 μm (**A**) and 500 nm (**B**).

Since the expression of autophagy genes and proteins was significantly increased in the cerebral cortex, we used TEM to assess if autophagic structures accumulate in cerebral cortical neurons in response to chronic RF-EMF exposure. Many autophagosomes and autolysosomes were evident in cerebral cortical neurons of the RF-EMF exposure group compared to the control group (Fig. 5A). Sequential formation of autophagosomes and autolysosomes were observed in cerebral cortical neurons of RF-EMF exposed mice. The extended double membrane of phagophores became mature autophagosomes, which engulfed intracellular organelles and then fused to lysosomes to form autolysosomes (Fig. 5B).

### Chronic RF-EMF exposure induces myelin damage of cerebral cortical neurons

**Figure 6:**
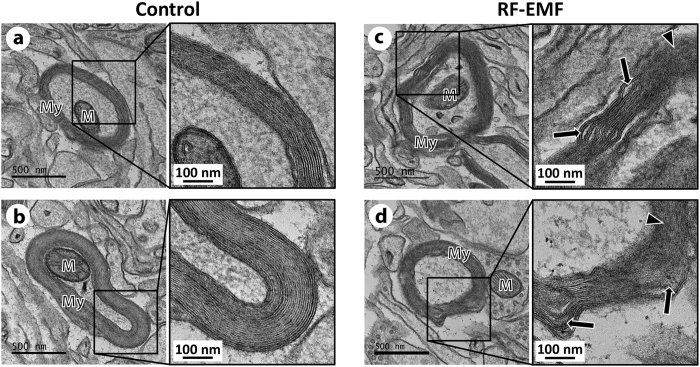
Ultrastructural alterations of myelin sheaths in neurons of the cerebral cortex in mice following chronic RF-EMF exposure. Representative TEM micrographs were acquired from sham control (**a**,**b**) and RF-exposed mice for 12 weeks (**c**,**d**), respectively. Right side figures showed high power images of each insert (black dotted box) in **a**–**d**. Allows indicates damage to myelin sheaths with unusual myelin protrusions into cortical neuron in mice following RF-EMF exposure. Also, allow heads show breakdown sheaths thereby showing blurry myelin. M, mitochondria; My, myelin sheaths. Size bars: 500 nm (Low power images) 100 nm (High power images).

The observations of autophagosome formation and down-regulation of pro-apoptotic factor Bax suggested a lack of neuronal damage. To explore the integrity of axons after the chronic RF-EMF exposure, myelin sheaths of axons in the cerebral cortical neurons were examined by TEM. Copious alteration of axon myelin sheaths in the cerebral cortical neurons was evident (Fig. 6c and d). The defective myelin sheaths had a frayed lamellae structure and unusual myelin protrusions, and were surrounded axon by blurry loops of myelin flanking into cortical neurons (Fig. 6c and d). Sham-exposed mice did not show any such changes (Fig. 6a and b). This result indicated that RF-EMF exposure for 12 weeks could cause myelin damage of the cerebral cortical neuron.

## Discussion

RF-EMF (835 MHz, 4.0 W/kg SAR, 5 hours a day for 12 weeks) rendered mice hyperactive, induced autophagy in the cortical cell body with significant increases in the transcriptional and protein levels of autophagy and accumulation of autophagosomes, and caused myelin damages in the cortical neurons.

The global burgeoning of mobile phone use has triggered concerns regarding potential harmful effects of RF-EMF exposure, given the evidence in animal models of deleterious neuronal changes RF-EMF exposure^5^,^6^,^7^, neurological and cognitive dysfunctions^9^,^10^, enhanced *in vitro* apoptosis^12^,^13^, and altered mammalian cell autophagy^17^.

Our prior report that autophagy is activated in the mouse brain^15^ prompted us to speculate that the neuronal system in the brain remains stable and capable of normal function. We explored this speculation in the present study by assessing whether 835 MHz RF-EMF exposure altered brain function and neurobehaviour in mice. RF-EMF exposure led to hyperactivity behaviour as apparent by significant increases in moving distance and duration in the open field test (Fig. 1c and d). In addition, RF-EMF did not affect the basic motor ability in the rota-rod test (Fig. 1a). Similarly, 800–1900 MHz RF-EMF exposure of fetal mice impaired glutamatergic synaptic transmission in the pyramidal neurons of the prefrontal cortex, causing hyperactivity and memory deficit in the mice after birth^36^.

We previously reported that exposure of mice to 835 MHz RF-EMF for 12 weeks induces autophagy in specific inner brain tissues^16^. In this study, we extend these findings by elucidating whether autophagy is also activated in the cerebral cortex, the most outer portion of the neuronal brain. RF-EMF exposure intensified the autophagic response, with augmented levels of most autophagic components (e.g., LC3 and Beclins) in the cerebral cortex compared to other brain regions examined in other studies. Being relatively more external, the cerebral cortex is more exposed to RF-EMF, with the induction of autophagy being more likely compared to more inner regions of the brain.

Autophagy is a catabolic cellular process that involves the degradation of injured cytoplasmic organelles or usual protein aggregates^35^. Autophagy-related genes used for RT-PCR are key factors for autophagosomal formation^35^. The process of autophagy is initiated by the formation of a precursor membrane termed the phagophore, which encircles targeted cytosolic organelles or proteins, and eventually forms a double‐membrane vesicle termed the autophagosome^37^. Autophagosome formation can be initiated via AMP-activated protein kinase (AMPK) activation, which phosphorylates Ulk1 kinase^35^. The activated Ulk1 kinase (UNC 51-like kinase 1) complex and a multi-protein complex containing Beclin1/2 and the class III phosphoinositol-3-kinase (PI3K CIII, also known as Vps34) complex drive vesicle nucleation. Transmembrane protein Atg9 may recruit lipid to phagophores^38^. In the elongation step, LC3 undergoes post-translational modifications in which LC3 is cleaved by the cysteine protease Atg4^39^. An E3 ubiquitin ligase-like enzyme Atg5 complexed with Atg12 and Atg16L1 regulates the formation of a complex involving microtubule-associated protein 1 light chain 3 (LC3)-I and phosphatidylethanolamine (PE) to form LC3-II (LC3-PE), which is critical for autophagic vesicle incorporation in the autophagosomal membrane^39^. RF-EMF exposure significantly augments in the protein level of LC3B-II, which is a widely used marker for autophagosomes in the cerebral cortex, which reflect the relative amount of autophagosomes in cerebral cortical neurons. Eventually, the mature autophagosome fuses with a lysosome to form an autolysosome^19^.

Exposure of rats to 900 MHz RF-EMF reportedly up-regulates the apoptotic pathway in the cerebellum^40^. Similar cellular stressors are involved in autophagy and apoptosis; they are both tightly regulated by inhibition of mediator proteins including Bcl2 and Beclin1^41^,^42^. Thus, we examined whether apoptotic cell death is activated together with the autophagic pathway, during which autophagy is activated in response to RF-EMF exposure. The expression level of both the transcripts and protein of Bcl2 and Bax was down-regulated in the cerebral cortex of mice (Fig. 4).

In this study, we found anti-apoptotic member and pro-apoptotic member are both decreased in the cerebral cortex after 835 MHz RF-EMF exposure. Basically, the decreased expression of pro-apoptotic Bax might attenuate apoptotic pathway and drive the cell to adapt for RF-EMF by autophagy formation in cortical neurons. However, the decrease in the Bcl2 protein levels might be induce apoptosis but simultaneously activate autophagy pathway. In previous study, Bcl2 could inhibit Beclin1 dependent autophagy in human breast carcinoma cells^41^. Therefore, the decreased Bcl2 might be consistent with the activation of autophagy pathway in our study, although the detailed underlying mechanisms are unclear yet. The RF-EMF exposure conditions may have imposed weak stress, which may have facilitated the activation of the autophagy pathway for initial adaptation from RF-EMF. However, if cortical neurons received stronger RF-EMF stress, cell death might result due to apoptosis of the cerebral cortical neurons. Further studies are needed to clarify whether long-term exposure (for more than 3 months) or other RF-EMF exposure conditions cause apoptotic cell death in neurons of brain tissues.

TEM examination of cell body neuronal structures revealed accumulation of autophagosomes and autolysosomes in the cerebral cortical neurons following the 12-week RF-EMF exposure. These neuronal changes are consistent with the up-regulated gene expression and protein production in the cerebral cortex after RF-EMF exposure. Furthermore, autolysosomes fused with autophagosomes and lysosomes were observed (Fig. 5B), indicating autophagy induction in the cerebral cortical neurons after the 12-week RF-EMF exposure.

The cerebral cortex processes most of the actual sensory functions and movement information, as well as cognitive functions, such as thought and perception^24^,^25^. Abnormalities of the cerebral cortical region have reported in Alzheimer’s disease (AD) and Lafora disease (LD), and may cause various cognitive disorders^26^,^27^,^28^. Importantly, AD and LD are caused mainly by impairment of autophagy, which leads to accumulation of causal protein aggregates, such as Amyloid-β and Lafora bodies, respectively^43^,^44^. However, the current data strongly suggest that autophagy is activated in the cortical neuronal cell body by RF-EMF exposure as a means of cell survival from stress or to avoid neuronal cell death that otherwise could cause neurodegenerative diseases. Therefore, autophagy protects neurons against chronic RF-EMF exposure.

Presently, 835 MHz RF-EMF exposure at a SAR of 4.0 W/kg for 12 weeks induced autophagy in neuronal cell bodies in the cerebral cortex of mice, perhaps to protect against chronic RF-EMF insult. Hence, most of the neuronal cell bodies might be structurally stable. Appropriately, we explored whether RF-EMF stress affected myelin integrity. Many myelin sheaths were damaged, with scarring and loose loops of myelin sheaths evident (Fig. 6). Myelin is a protective layer of fatty white matter that surrounds the axon in the nerves of the nervous system. Myelin acts as an electrical insulator for transmission of electrical signals in the nervous system^45^. Damage to the myelin sheath (demyelination) commonly results in multiple sclerosis (MS) which causes inflammation and injury to the sheath^46^. MS is unusual for children but severe symptoms in children include altered consciousness, confusion, and visual impairments^33^. Also, exposure to RF-EMFs may affect myelin damage and lead to functional impairment with symptoms of hypersensitivity in humans^33^. The latter authors reported an association between RF-EMF and myelin alterations, as well as common symptoms between electro-hypersensitivity and MS caused by demyelination^33^.

To explore the possible biological effects of RF-EMF exposure, we used 4.0 W/kg SAR value for animal experimental model in our study. Also, Switzer and Mitchell (1977) previously reported that continuous 2450 MHz RF-EMF exposure could increase the myelin degeneration of neurons in the brain of rat. This result strongly suggests that even the lower levels of exposure of RF-EMF could cause for the myelin lesions^47^. The International Commission on Non-Ionizing Radiation Protection (ICNIRP) guideline was suggested for providing adequate protection for EMF exposure. In the safety level, 4.0 W/kg SAR value is the maximum permitted exposure to human limbs for general public person and the number could go up to even 20 W/kg for occupational exposure. In the localized SAR, head and truck are 2.0 W/kg for general public and 10 W/kg for occupational exposure. Therefore, there is a possibility that the brain of living creatures might be exposed at the higher amounts of EMF than 4 W/kg even in safety level.

In terms of a possible thermal effect on body, we confirmed that exposure to RF-EMF in our system could not affect mouse body temperature as shown in Fig. S2. This may be contributed to body temperature regulatory mechanism as well as mouse moving freely. Following information for thermal effect of high frequency (100 kHz–300 GHz) on body from ICNIRP, the body has a regulatory system for the internal temperature. If the body temperature rises to an unacceptable level, it will cause serious problems such as heat stroke and tissue damage. Thus, exposure to RF-EMF could have the small thermal effect but not lead to thermal damage in our RF-EMF exposure system because the body has a control system to an increase in temperature in a small range. Importantly, the blood circulation in the brain can dispose of excess heat by increasing local blood flow. Free Moving is important issue because when RF-EMF is exposed by immobilized condition with special apparatus or anesthetization, this immobilized exposure could result in higher thermal effect on body. There are few reports resulting in thermal effect or thermal damage to anesthetized rats after magnetic field (MF) or RF exposure, respectively^48^,^49^. However, in our RF exposure system, mice can move freely in their cage inside RF-EMF generator during exposure. Rodent studies by RF-EMF exposure have showed high value of SAR exposure could not produce big change of body temperature which was within the normal range of body temperature^50^,^51^. In their condition, animals can move freely in cages during exposure. Our RF-EMF system could provide that RF-EMF exposure at 4.0 W/kg could not lead to thermal damage to mice body. Therefore, the results including the alterations of myelin sheaths in the cortical neuron are less likely coming from thermal damage in our study.

Whether myelin sheath disruption causes behavioural changes was explored. Mice exposed to the RF-EMF regimen displayed neuronal impairment evident as demyelination of cortical neurons, which might have influenced the hyperactive behaviour that was observed. Further research should explore whether RF-EMF exposure of fetuses exacerbates demyelination and eventually leads to altered behaviour with neurological dysfunctions by behavioural tests. Myelination from fetal to first year of life in humans is important for proper function of the brain^52^.

In summary, 835 MHz RF-EMF at 4.0 W/kg SAR for 5 hours daily for 12 weeks led to hyperactivity behaviour in mice. The autophagy pathway was activated but apoptosis in neuronal cell bodies was not. Myelin sheaths of axons were damaged in cerebral cortical neurons. The results may provide insight into the initial adaptation to RF-EMF stress including a protective process initiated in neuronal cell bodies in the cerebral cortex, which may be beneficial for maintaining a homeostasis of brain. Disrupted myelin sheaths might be a potential cause of neurobehavioural disorders.

## Methods

### Mice

C57BL/6 male, 6-week-old mice weighing 25–30 g were purchased from Daehan Bio Link (DBL, Chungbuk, South Korea). The mice were housed under specifically controlled conditions (ambient temperature of 23 ± 2 °C, 12-h light/dark cycle). Food pellets (Daehan Bio Link, Chungbuk, South Korea) and water were supplied *ad libitum*. After a 7-day adaptation period, mice were randomly assigned to a 12-week period of sham-exposure or RF exposure. All procedures were reviewed by the guidelines of the NIH for animal research and were approved by Dankook University Institutional Animal Care and Use Committee (IACUC; DKU-15-001), which adheres to the guidelines issued by the Institution of Laboratory of Animal Resources.

### RF-EMF exposure

Mice were exposed to 835 MHz RF-EMF using a Wave Exposer V20 as described in detail^5^,^16^. Our research group had already tested and described about dosimetry for our RF-EMF generator previously, but we further examined and showed in detail (Supplementary Fig. S1). Firstly, we confirmed that RF-EMF generator created 835.367 MHz signal by measuring spectrum analyzer (NS-30A) (LIG Nex, Gyeonggi-do, South Korea). Subsequently, SAR value was estimated to be 4.0 W/kg by 0.0001°C resolution temperature sensor by measuring temperature changes of saline water of the mouse phantom exposed to 835 MHz of continuous wave (CW) without modulation. Temperature change of saline water was measured by 0.0001°C resolution in this research to obtain more precise SAR value with a finer temperature measurement system. SAR value in the central position of the mouse phantom was also acquired by numerical analysis by Ansys HFSS 13. Also SAR was evaluated by measuring E-field at the phantom position in the air and by considering the ratio of E-field in the liquid to E-field in the air at the same position in the same environment. Following calculation of SAR, the value is 4.14 W/kg which is proximity of setting up output power at SAR 4.0 W/kg of horn antenna of the exposure apparatus. As a results, our measurement of RF signal and SAR value generated from our RF-EMF generator produce 835 MHz RF-EMF with 4.0 W/kg SAR. Whole body exposure was at a SAR value of 4.0 W/kg for 5 h daily for 12 weeks for six randomly allocated mice. The other six mice received sham treatment for 12 weeks. The sham treated control groups were kept under the identical environmental conditions and treated the same circular pattern as the RF-exposed groups without RF-EMF exposure. The sham-treated and RF-exposed mice could move freely in their cage. The cage inside RF-EMF generator was 43 cm long × 37 cm wide × 18 cm high. RF-EMF exposure was a top horn antenna to the lower mouse cage. The bottom and wall of the cage were covered by ceramic wave absorption material. The intent was to mimic RF with SAR exposure in the open environment, to exclude the possibility of the influence of the number of mice on exposure. Importantly, the RF exposure apparatus was equipped with automatic light system, air conditioning, and water dispenser. The mice were not restricted in movement in the cage during the exposure. All the experiments have done in our animal facility, which were maintained in constant temperature.

### Rota-rod test

We examined experimental mice with Rota-rod behavioural test at the ends of the 12 week exposure of RF-EMF with eight sham-exposed mice and seven RF-EMF exposed mice. Mice were evaluated for their ability to stay on a rota-rod (Ugo Basile, Comerio VA, Italy) for at least 600 seconds (accelerated speed). Rota-rod testing was performed three times per mouse with an intervening interval of at least 10 minutes. The times in seconds from the three tests were averaged.

### Open field test

We examined the open field test with eight sham-exposed mice and seven RF-EMF exposed mice in one week after 12 week exposure of RF-EMF. There are nine plastic rectangular box for open field test, which can test nine mice’s behaviour simultaneously. Each mouse was located in a white plastic rectangular box 40 cm in length × 27 cm in width × 27 cm in height after RF-EMF exposure for 12 weeks. Locomotor activity of mice was monitored for 30 minutes in normal lighting using a CCD camera connected to an EthoVision Version 2.3 automated video recording and tracking system (Noldus Information Technology Inc., The Netherlands). Moving distances and durations, and rearing frequencies were calculated. The box was cleaned with 70% alcohol and water between trials to remove any stuffs which could affect the mouse behavioural patterns for next test or another batch of test.

### RNA purification and RT-PCR

Experimental mice were euthanized by cervical dislocation and head was quickly decapitated with scissors, then the cerebral cortex was rapidly dissected from each brain on ice. Total RNA was purified from the whole cerebral cortex of each mouse in each group using TRIzol reagent (Thermo Fisher Scientific, USA). RNA was reverse transcribed to cDNA using MMLV Reverse-Transcriptase (Bioneer, South Korea) and an oligo-d(T)18 primer. Quantitative RT-PCR reactions were performed with Rotor-gene SYBR Green supermix kit (QIAgen, USA) and fluorescence was measured using Rotor Gene PCR Cycler (QIAgen). The expression levels of the genes were normalized to that of glyceraldehyde 3-phosphate dehydrogenase (GAPDH) as a housekeeping gene. GAPDH primer was purchased from QIAgen. The primers used for qRT-PCR and sqRT-PCR (Table 2) were synthesized by Bioneer or Cosmogenetech (South Korea). Three biologically independent experiments were performed and each PCR reaction was done in triplicate. The relative levels of specific mRNA were calculated by normalization to GAPDH expression by the 2^−ΔΔCt^ method (n = 6). Also, the expression values of the RF-exposed groups were normalized to those of the sham-exposed group. Semi-quantitative RT-PCR reactions (sqPCR) were carried out using PCR PreMix (Bioneer). Subsequently, the sqPCR product of each gene was separated by 1.5% agarose gel electrophoresis and the signal intensity of each PCR product was visualized following staining of DNA using Syto 60 (Li-Cor, USA) using an Odyssey infrared imaging system (Li-Cor).

### Western blotting

Sham-or RF-exposed mice were quickly sacrificed and the cerebral cortex was rapidly dissected from each brain. Some tissue was lysed with RIPA lysis buffer (ATTO, Japan) supplemented with protease inhibitor and phosphate inhibitor cocktail (ATTO). Lysates were homogenized and sonicated briefly on ice. Concentration of proteins was estimated using a DC^TM^ protein assay (Bio-Rad, USA). Total protein (50–100 μg) was subjected to sodium dodecyl sulfate-polyacrylamide gel electrophoresis and the resolved proteins were transferred with EzFastBlot transfer buffer (ATTO) to a polyvinylidene difluoride (PVDF) transfer membrane (ATTO). Protein bands were visualized using C-DiGit Chemiluminescence Western Blot Scanner (Li-Cor). The intensity of band was quantified and normalized using α-tubulin as an internal control. Alterations in the expression level of autophagy-related proteins was further analysed with two-dimensional (2D) gel electrophoresis using a PROTEAN i12 IEF System (Bio-Rad).

### Transmission electron microscopy (TEM)

Other samples of the dissected cerebral cortex were fixed immediately in 2% glutaraldehyde and 2% paraformaldehyde in 0.1 M phosphate buffer (pH 7.4) for 2 hours at 4 °C. Following three washes in phosphate buffer, the brain tissues were post-fixed with 1% osmium tetroxide on ice for 2 hours and washed three times in phosphate buffer. The tissues were then embedded in Epon 812 mixture after dehydration in an ethanol and propylene oxide series. Polymerization was conducted with pure resin at 70 °C for 24 hours. Ultrathin sections (~70 nm) were obtained with a MT-X ultramicrotome (RMC, USA). The sections were collected on 100 mesh copper grids. After staining with 2% uranyl acetate (15 minutes) and lead citrate (5 minutes), the sections were visualized by TEM using a Technai G^2^ Spirit Twin apparatus (FEI, USA) at 120 kV.

### Statistics

All data are presented as the mean ± SEM from 6 mice in each experiment. The significance for all pairwise comparisons of interest was assessed by two-tailed Student’s *t*-test with probability values of P < 0.05 considered significant. We used GraphPad Prism 4 program (GraphPad Software, Inc., La Jolla, CA) for the statistical analysis.

## Additional Information

**How to cite this article**: Kim, J. H. *et al*. Long-term exposure to 835 MHz RF-EMF induces hyperactivity, autophagy and demyelination in the cortical neurons of mice. *Sci. Rep.* **7**, 41129; doi: 10.1038/srep41129 (2017).

**Publisher's note:** Springer Nature remains neutral with regard to jurisdictional claims in published maps and institutional affiliations.

## Supplementary Material

Supplementary Information

## Figures and Tables

**Table 1:** Average fold-changes of the expression level of autophagy-related genes in the cerebral cortex following 835 MHz RF exposure for 12 weeks.

Genes	Fold change	
	Sham	RF
AMPK1α	1	1.20
Ulk1	1	1.34
Beclin1	1	1.49
Beclin2	1	2.33
Atg9A	1	1.51
Atg4A	1	2.69
Atg4B	1	1.54
Atg5	1	1.64
LC3A	1	1.31
LC3B	1	2.40

Expression of autophagy genes was analysed by qRT-PCR. The relative mRNA levels of genes were calculated by normalizing to expression of GAPDH by the 2^−ΔΔCt^ method.

**Table 2:** Oligonucleotide sequences of RT-PCR primers^16^.

Gene	Forward (5′ → 3′)	Reverse (5′ → 3′)
AMPK1α	TCAGACCCTCCAAGCCCTGAATTT	TACAGTGTGAGCAACTCGGCTCTT
Ulk1	ATCATGTTGCTCTGGAGGTA	AAGACACTGTCAGGCTTTTC
Beclin1	CTGAAACTGGACACGAGCTTCAAG	TGTGGTAAGTAATGGAGCTGTGAGTT
Beclin2	ATTTCAGATGAGGGTCCCTTG	CAAGGACTTGAGATAGGAATGG
Atg9A	TCATGCAGTTCCTCTTTGTGG	TCTGGCAGAGTGACCTTG
Atg4A	CCCTCACACAACCCAGACTT	CCCCTGTGGTTGTCACTTCT
Atg4B	ACGGAGGAAGACTTTAACGAC	AAACCTCTCCAGTCTCTCTAC
Atg5	GGAGAGAAGAGGAGCCAGGT	TGTTGCCTCCACTGAACTTG
LC3A	TGGTCAAGATCATCCGGC	CTCACCATGCTGTGCTGG
LC3B	TTCTTCCTCCTGGTGAATGG	GTGGGTGCCTACGTTCTCAT
Bcl2	ACCGTCGTGACTTCGCAGAG	GGTGTGCAGATGCCGGTTC
Bax	CGGCGAATTGGAGATGAACTG	GCAAAGTAGAAGAGGGCAACC
