# Supplementary material for: Long-term exposure to 835 MHz RF-EMF induces hyperactivity, autophagy and demyelination in the cortical neurons of mice
Source: Sci Rep. 2017 Jan 20;7:41129. doi: 10.1038/srep41129 (PMC5247706; doi:10.1038/srep41129)
Supplement: Supplementary Information [file srep41129-s1.doc]

**Long-term exposure to 835 MHz RF-EMF induces hyperactivity, autophagy and demyelination in the cortical neurons of mice**

Ju Hwan Kim^1^, Da-Hyun Yu^1^, Yang Hoon Huh^2^, Eun Ho Lee^1^, Hyung-Gun Kim^1^, Hak Rim Kim^1^†

*^1^Department of Pharmacology, College of Medicine, Dankook University, Cheonan-si, Chungnam, Republic of Korea.*

*^2^Center for Electron Microscopy Research, Korea Basic Science Institute, Ochang, Chung-Buk, Republic of Korea.*

Running title: Induction of autophagy and demyelination in cerebral cortex

† Correspondence and Proofs: Hak Rim Kim, Ph.D.

Department of Pharmacology

College of Medicine, Dankook University

119 Dandaero, Cheonan

Chungnam, 31116 ROK

Tel: +82-41-550-3935

Email: hrkim@dankook.ac.kr


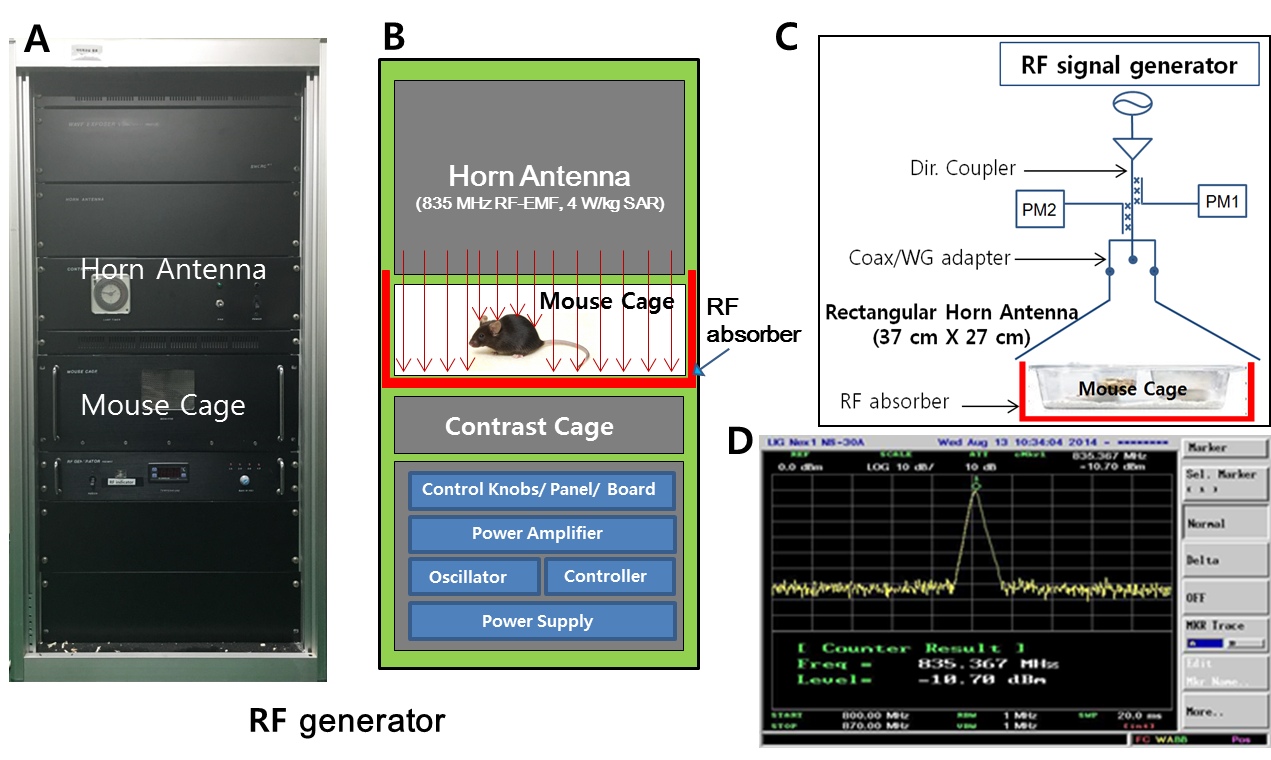
**Supplementary information**

**Fig. S1. Radiofrequency exposure apparatus (Wave Exposer V20) is used for RF-EMF exposure of mice to 835 MHz at 4.0 W/kg SAR.** (A) An exterior view of RF generator. (B) Schematic outline in RF generator. (C) System block diagram of horn antenna and mouse cage. (D) Measured 835 MHz radiofrequency radiation generated from RF generator by spectrum analyzer (NS-30A).

**SAR measurement in a liquid mouse phantom exposed by 835 MHz rectangular horn antenna system.**

RF exposure system (Fig S1 A-C) was generated to investigate the effect of radiation on mouse at 835 MHz of continuous wave (CW). This RF-EMF instrument can set up four different SAR values (1.6, 2.0, 3.0, 4.0 W/kg) but we only used the value of 4.0 W/kg SAR exposed to mice in this experiment. Thus, to estimate the correct dose of the experimental animals, we measured exact RF signal radiation and SAR value generated from RF generator.

Firstly, we confirmed whether RF generator is created 835MHz signal. To do this, we estimated RF signal with spectrum analyzer (NS-30A) (LIG Nex, Gyeonggi-do, South Korea). We confirm that the RF-EMF instrument generator generates 835.367 MHz RF-EMF signals (Fig S1 D). Subsequently, SAR value was estimated to be 4.0W/kg by 0.0001˚C resolution temperature sensor by measuring temperature changes of saline water of the mouse phantom exposed to 835 MHz of CW. Temperature change of saline water was measured by 0.0001℃ resolution in this research to obtain more precise SAR value with a finer temperature measurement system. SAR value in the central position of the mouse phantom was also acquired by numerical analysis by Ansys HFSS 13. Also SAR was evaluated by measuring E-field at the phantom position in the air and by considering the ratio of E-field in the liquid to E-field in the air at the same position in the same environment.

**SAR measurements.**

SAR (specific absorption rate) at a point in the lossy medium exposed by high frequency electromagnetic field is defined in eq. (1). (IEEE Std 1528TM-2013, IEEE Recommended Practice for Determining the Peak Spatial-Average Specific Absorption Rate (SAR) in the Human Head from Wireless Communications Devices: Measurement Techniques, IEEE Standards Coordinating Committee 39, IEEE).\

Specific heat capacity of the saline water C is 4,140 [J/kg ˚C], while electrical conductivity of the medium σ was 2.044 [S/m]. mag is electric field intensity in magnitude of phasor[V/m], mass density ρ of 0.9 % saline water is 1,002 [kg/m3] at 20˚C, but it is set to be 1,000 [kg/m3] in usual SAR measurements. T is temperature (˚C) of 0.9 % saline water, and t is duration time of 835 MHz RF exposure. The changes of saline water’s temperature (˚C) following different exposure time of 835 MHz RF showed in Fig 3. SAR value was calculated by the conditions that ΔT = 2.1˚C and Δt = 3600 sec. SAR = 4.14W/kg in this case. Following calculation, the value is 4.14W/kg which is proximity of setting up output power at SAR 4.0 W/kg of horn antenna of the exposure apparatus. As a results, our measurement of RF signal and SAR value generated from our RF generator produce 835 MHz RF-EMF with 4.0 W/kg SAR.

**Measurement of mice body temperature before/after RF-EMF exposure**

RF-EMF can generally penetrate into the body and vibrate molecules which can generate heat in the body. To confirm whether exposure to RF-EMF signal affect mouse body temperature in our system, actual mice body temperature was measured by using a special temperature measuring instrument, testo 925 (Kalibrier-Protokoll, Germany). Each mice group includes in 8 mice which inserted a mouse rectal probe to a depth of 1.5 cm of the mouse's anus. We measured their body temperature three times (once a week for 3 weeks) before sham or RF-EMF exposure, right after 5 hours sham or RF-EMF exposure and 2 hours after 5 hours sham or RF-EMF at 4.0 W/kg SAR exposure. The result showed that mice body temperature did not alter after RF-EMF exposure compared to sham-exposed mice (Ctrl) (Fig. S2).

The results indicated that mouse body temperature was not significantly affected following exposure to 835 MHz RF-EMF at 4.0W/kg SAR for 5h/day emitted from our RF-EMF generator. Therefore, the thermal damage is hardly happening in our study.


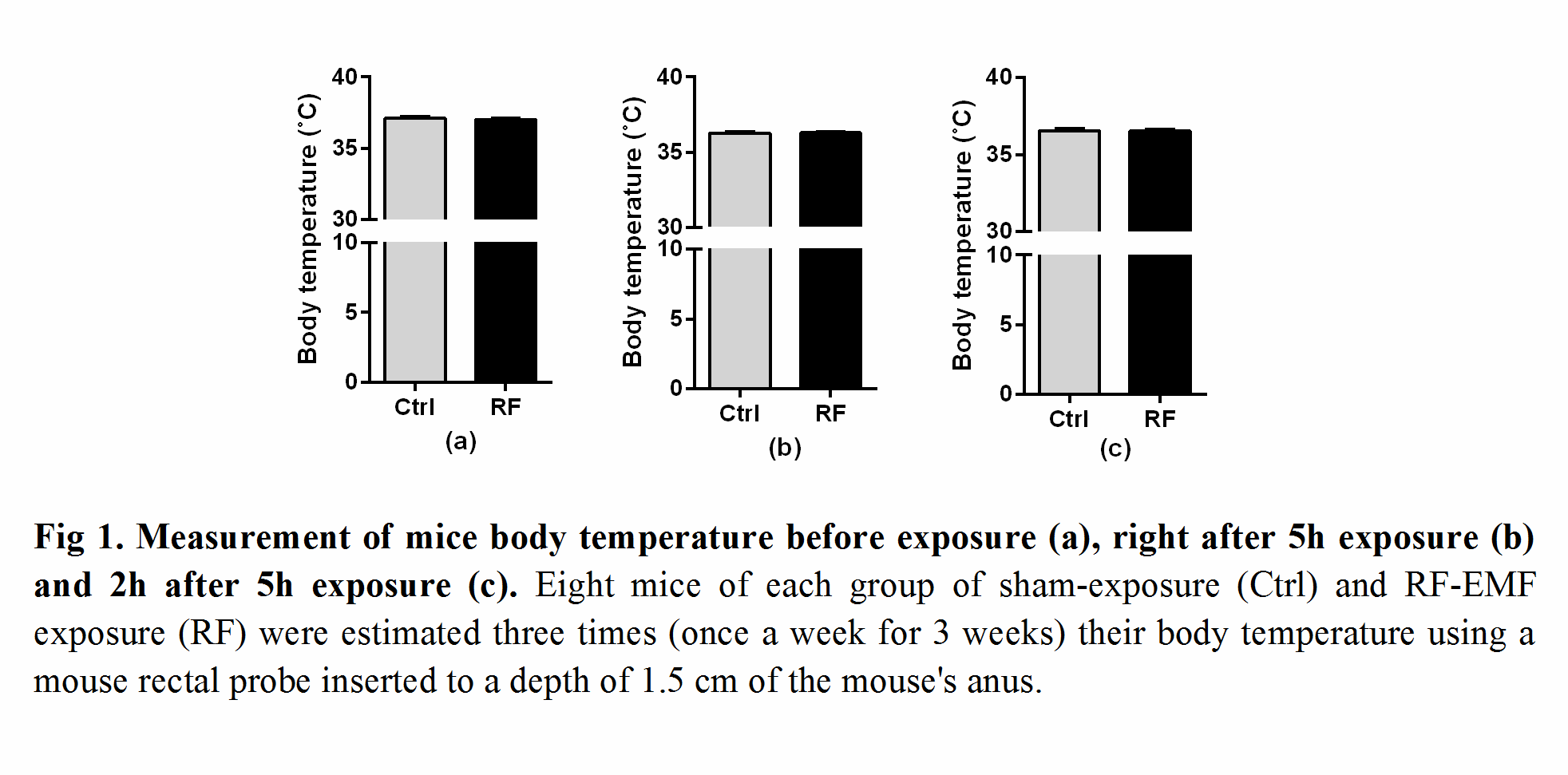


**Fig. S2. Measurement of mice body temperature before exposure (a), right after 5h exposure (b) and 2h after 5h exposure (c).** Eight mice of each group of sham-exposure (Ctrl) and RF-EMF exposure (RF) were estimated three times (once a week for 3 weeks) their body temperature using a mouse rectal probe inserted to a depth of 1.5 cm of the mouse's anus.
